# Suicide Risk Assessment in Australian Emergency Departments: Assessing Clinicians' Disposition Decisions

**DOI:** 10.1155/2014/943574

**Published:** 2014-04-07

**Authors:** T. J. Weiland, A. Cotter, G. A. Jelinek, G. Phillips

**Affiliations:** ^1^Emergency Practice Innovation Centre, St. Vincent's Hospital Melbourne, Fitzroy, VIC 3065, Australia; ^2^Faculty of Medicine, Dentistry and Health Sciences, The University of Melbourne, VIC 3010, Australia; ^3^Department of Epidemiology and Preventive Medicine, Monash University, Melbourne, VIC 3800, Australia; ^4^Crisis Assessment & Treatment Team, St. Vincent's Hospital Melbourne, Fitzroy, VIC 3065, Australia

## Abstract

*Objective*. To determine (1) the uniformity of disposition decisions made by clinicians working in Australian emergency departments (EDs) using vignettes describing patients presenting with deliberate self-harm or suicide risk; (2) factors associated with these decisions; (3) factors associated with confidence in these decisions. *Methodology*. We validated and distributed by email an online survey tool to Australian emergency clinicians via their colleges. Participants were presented with five vignettes and asked to rate the level of risk and protective factors for suicide, the patient's disposition (admit/discharge/review), factors influencing this decision, their confidence in the decision, and factors that would have improved their confidence. *Results*. Percentages of participants choosing the modal disposition decision for each scenario ranged from 58.6% (136/232) to 92.4% (220/238), demonstrating uniformity in clinicians' disposition decisions. Predictors of disposition were consistently level of risk factors perceived and, infrequently, clinician factors including age and years experience. Confidence in disposition decisions was high across scenarios. Clinicians reported patient, clinician, contextual and decision support factors relevant to an Australian emergency context affected their disposition decisions and confidence in decisions. *Conclusion*. Emergency clinicians are uniform and confident in their disposition decisions for patient vignettes where there is risk of suicide or self harm.

## 1. Introduction

Suicide is a major health concern, accounting for 2361 deaths (suicide rate: 10.6/100,000) in 2010, making it the fifteenth overall leading cause of death in Australia [1]. Attempted suicide or suicidal ideation is amongst the most common mental health issues likely to require emergency department (ED) management [2], due to the urgency of the situation and also, in part, due to a lack of alternate services outside normal working hours [3]. EDs are the primary access point to hospital care and play a key role in sorting and referring patients at risk of suicide for psychiatric follow-up [3]. Consequently, EDs have a unique role as a “first port of call” for the identification of patients at risk of suicide. Accurate, consistent, and timely managed decisions regarding these patients are critical to outcome. These features of EDs, combined with the fact that a high proportion of those that self-harm and complete suicide present to a healthcare provider not long before their attempts [4], suggest that there is scope for EDs to reduce deaths through effective identification and appropriate management of at-risk individuals [5].

A systematic review of suicide risk assessment and management by the Victorian Government Department of Health indicated that single factors were not predictive of patient suicide risk [4], necessitating clinician consideration of the complex interplay of multiple factors in suicide risk assessment. There is, necessarily, a heavy reliance on clinician judgment since existing tools lack the necessary balance of sensitivity and specificity [4, 6] to safely rule out those without risk. Many tools have been developed for the assessment of deliberate self-harm risk but few are used clinically, with clinicians citing problems such as the time taken to administer them. In any event, these tools have been shown to be as accurate as a clinician's global assessment [7]. Clinician judgments should ideally take into consideration past clinical experience, the current clinical presentation including observations of patients' general appearance, affect, behaviour, and conversation [8–13], and the assessment and management options available at a particular health service [5]. An intelligent integration of assessment scales with a wide knowledge and qualitative clinical judgment by the assessing clinician is recognized as an effective method for determining suicide risk assessment [8–13], using risk assessment tools as a framework to ensure comprehensive and systematic questioning about suicide risk [5]. Following risk assessment, a “disposition decision” is made by the clinician, either to admit the patient for inpatient services or to discharge the patient and refer for review at a later stage.

The increasing numbers of patients at risk of suicide presenting to Australian EDs and the high rates of suicide in Australia highlight the need for clinicians to be able to appropriately assess and manage these patients. It is therefore important to assess the degree to which clinicians can adopt uniform assessment and management decisions for patients at risk of suicide in the context of current Australian patient populations, clinician training, guidelines, and the scope of health services available.

Past research has shown that factors such as patient populations, clinician training and guidelines, hospital characteristics, and the scope of health services may influence decisions made about management of patients at risk of suicide [14]. Given the unique nature of each of these factors in an Australian ED setting, the identification of factors that Australian emergency clinicians consider when making a disposition decision is warranted.

We have previously demonstrated that Australian ED clinicians report a lack of confidence and training in assessing and managing suicide related presentations [15, 16]. The identification of factors contributing to lower levels of confidence may be helpful in developing targeted training programs that address these issues, as clinicians are increasingly called upon to assess and manage suicide risk related presentations in the ED.

For patients presenting with deliberate self-harm with or without suicidal intent, we aimed to (1) quantitatively assess the uniformity of disposition decisions made by Australian emergency clinicians following assessment of patient suicide risk, (2) explore factors affecting their disposition decisions, (3) investigate levels of self-reported staff confidence in disposition decision making, and (4) explore factors which may improve staff confidence in disposition decision making.

## 2. Method

### 2.1. Design and Participants

We undertook a cross-sectional survey of a national sample of nurses and doctors working in Australian EDs. All members of CENA (College of Emergency Nursing Australasia) and ACEM (Australasian College for Emergency Medicine) working clinically in an Australian ED were eligible for participation in the study. There were no exclusion criteria.

### 2.2. Survey Tool

We adapted an existing survey tool [16] which was originally developed according to Lynn's method for instrument development [17] and included nine vignettes from which five were selected for the current study (Table 4). Vignettes have been demonstrated in the literature to produce comparable clinician responses as in live case presentations [18, 19]. The vignettes included three scenarios in which risk and protective factors were balanced, as well as the high and low risk test scenarios designed to polarize responses. Each vignette described a typical clinical presentation where an assessment of suicide risk would be required. In accordance with the literature, three major categories of presenting diagnoses were represented, psychotic disorders, affective (mood) disorders, and other disorders such as personality disorders. Vignettes also included information relating to five key factors associated with suicide risk, social connectedness, intellectual functioning, previous suicide attempts, situational crisis, and diagnosis [20].

The survey consisted of 38 items: eight general demographic questions about participants (age, gender, years of working clinically in an ED, and role in ED; hospital type (metropolitan/regional-rural) and region of practice; whether the hospital that the participant practiced in had an ED accredited by the ACEM for emergency medicine training; patient types treated (children only/mixed/adults only)) and six identical items for each of the five vignettes; (1) a rating of risk; (2) protective factors for the patient; (3) a disposition decision for the patient (admit/discharge for community team review within 24 hours/discharge, for general practitioner review within 7 days); (4) factors considered in the disposition decision; (5) a rating of the level of confidence in the decision; and (6) factors which would improve the level of confidence if confidence was low.

For items requiring a rating, a one-to-ten interval scale was used. Endpoint descriptors were used (e.g., “1 = low level risk” and “10 = high level risk”) for the items requiring a rating of patient risk factors, patient protective factors, and confidence in making a disposition decision.

Open-ended questions requested participants to reflect on other factors they considered in making the disposition decision for each scenario, and for suggestions of what would make them more confident in the decision for each scenario, if confidence was low.

### 2.3. Content Validation

For content validation, two doctors and four nurses working clinically in the ED at St. Vincent's Hospital (Melbourne) completed a pilot survey to assess the relevance of the survey tool. Participant ratings of item relevance were used to calculate the content validity index of survey items [17], and survey items were deemed valid. Participants were also invited to comment on the content and wording of individual items. The wording of two items was modified based on this feedback.

### 2.4. National Survey

The survey was distributed to nurses and doctors electronically, via their professional bodies, CENA and ACEM, respectively. An introductory email was sent inviting all members working clinically in Australian EDs to participate in the study. A plain language statement advised that partial or full completion of the study would be taken as implied consent to participate in the research. The email included a hyperlink to the electronic questionnaire, hosted by the online survey package, “Survey Monkey.” The results of the questionnaire were collected over a period of six weeks, with one reminder email, identical to the original email, sent a week after the initial email. Participation required approximately 25 minutes.

### 2.5. Sample Size Calculation

Sample size calculations were based on member numbers provided by ACEM: 1340 fellows and 1984 trainees (17/2/11), and CENA: 1037 nurses (15/2/11). Using an online sample size calculator [21], a minimum sample of 354 emergency clinicians was required for a 5% margin of error and a 95% confidence interval, assuming a 50% response distribution.

### 2.6. Data Analysis

Data were analysed using SPSS version 18 (Chicago, IL). Summary statistics (percentage, 95% confidence intervals, and medians) were calculated for each item on the survey for the whole sample. Prior to comparing the disposition outcome by demographic data, the disposition item was recoded from three categories (admit, discharge for community team review within 24 hours, or discharge for GP review within 7 days) to two categories, admit or discharge, since very few respondents selected community team review as the disposition. Age was recoded into groups: 20–29, 30–39, 40–49, and 50 or more years. Similarly, years experience in ED was recoded into two categories: 1–5 years of experience and 6 or more years of experience. Role in ED (FACEM, advanced trainee, trainee, registered nurse, clinical nurse-specialist/educator/consultant, nurse unit manager, nurse practitioner, career medical officer, and other) was dichotomised into doctor or nurse, and patient population (adults only, children only, or mixed) was collapsed into two groups: adults only or children/mixed.

Analysis of ordinal data from items using the 1–10 interval scales was performed using summary statistics for each item (mean, median). For the purposes of data analysis, an a priori decision was made by the researchers to consider median scores of less than 4/10 as “low,” scores between 4/10 and 6/10 inclusive as “moderate,” and scores of greater than 6/10 as “high.”

For the primary outcome of interest, uniformity of disposition decisions, 95% confidence intervals were calculated for the modal disposition decision for each scenario. Disposition decisions were considered uniform where the lower and upper bounds of the 95% confidence interval for percentage of participants choosing the modal disposition did not include 50%. Scenarios in which the upper and lower bounds of the 95% confidence interval for percentage participants choosing the modal disposition overlapped with 50% were not considered uniform.

Analyses of nominal data from the dichotomous disposition decision item were performed using Fisher's Exact Test for 2 × 2 contingency tables or Chi Square where appropriate, to compare effects of gender, age group, years experience, role in ED (doctor or nurse), hospital type (metro. or regional-rural), or patient population (adults only or children/mixed). A Mann-Whitney *U* test was performed to investigate the association between the dichotomous disposition decision and participant ratings of perception of patient risk to self and others, protective factors mitigating risk factors, and confidence in disposition decision.

Logistic regression was used to identify predictors of disposition decision for each of the five scenarios using the enter method. For each model seven predictors were included using the enter method: staff type (Dr./nurse); staff gender (M/F); staff age (continuous); ED type (adult/mixed); years experience (continuous); staff rating of risk factors (0–10); staff rating of protective factors (0–10). Preliminary tests of the assumptions of logistic regression were performed, including an examination of multicollinearity to ensure that continuous independent variables were not closely correlated (being a bivariate correlation >0.70). Goodness of fit was assessed.

For all inferential tests, alpha was set at 0.05 and two-tailed tests of significance were used.

### 2.7. Ethics Approval

Ethics approval was granted by the host institution Human Research Ethics Committee, St. Vincent's Hospital (Melbourne). Additionally, endorsement was granted from the Australasian College for Emergency Medicine and College for Emergency Nursing Australasia, through their Scientific Committee review processes.

## 3. Results

### 3.1. Participation

Of 364 survey respondents, 30 indicated that they were not currently working in a clinical role in an Australian emergency department, excluding them from the research. This produced a final response rate of 334/4361 (7.7%), comprising 81 CENA members, 147 ACEM members, and 106 unspecified respondents.

### 3.2. Demographics

The majority of respondents worked clinically in EDs in Victoria (32.3%), New South Wales (23.6%), or Queensland (14.8%), and 72.5% (166/229) indicated that they worked in EDs in areas considered metropolitan rather than regional-rural. Almost all participants reported working in EDs accredited for training (208/226, 92.0%), with uncertainty about this amongst nine nurses only. Respondents mainly worked in hospitals treating a mixture of adults and children (163, 71.2%) followed by adults only (62, 27.1%) then children (4, 1.7%).

Around two-thirds of respondents were associated with ACEM (147/228, 64.5%), as either a Fellow of the Australasian College for Emergency Medicine, advanced trainee, trainee, or career medical officer. Over one-third of respondents were associated with CENA (81/228, 35.5%), either as a registered nurse (33/228, 14.5%), clinical nurse (specialist/educator/consultant) (36/228, 15.8%), nurse unit manager (5/228, 2.2%), nurse practitioner (5/228, 2.2%), or in other roles. Two respondents recorded other roles in ED, one being a nurse practitioner candidate and the other an associate nurse unit manager.

The median age of respondents was 38 years (IQR: 32–46), and just over half of the survey respondents were female (120/230, 52.2%). Respondents recorded a median of 9 years (IQR: 5–15) of experience in a clinical role in ED.

### 3.3. Suicide Risk Assessment and Uniformity of Disposition Decision

Respondents rated all scenarios as having a high level of patient risk of suicide, with the exception of scenario 2 which was respondent-rated as low risk (Table 1). Consistent with this, disposition decisions favoured admitting the patients in proportion to the perceived height of risk, except the patient in scenario 2. The uniformity of disposition decisions was good with no confidence intervals overlapping the preset criteria of 50% (Table 1). Perceived protective factors varied across scenarios and tended to parallel disposition decisions, the scenarios with low to moderate levels of protective factors, resulting in an admit decision by the majority of respondents.

### 3.4. Confidence in Disposition Decision

Confidence in all disposition decisions was high for all scenarios (Table 1), and for those scenarios where the modal decision was to admit admission was significantly associated with increased confidence (Table 2; *P* < 0.001 for scenarios 1, 3, 4, and 5).

### 3.5. Predictors of Admission

The most common independent predictor of the disposition decision to admit was the suicide risk rating assigned by the clinician; this was a significant factor for all five scenarios (Table 3), being strongest for scenario 4, wherein the odds of admission increased six times for each increase of one point on the risk rating scale. Protective factors were significant independent predictors for two scenarios, scenarios 3 and 5; in both instances a negative association was observed with lower ratings being associated with an admission decision (Table 3). Staff factors were less commonly observed as independent predictors of the decision to admit with staff age being associated with admission for scenario 3, with lower age raising the odds of admission slightly, and years experience in ED being associated with the decision to admit in scenario 4 (Table 3), wherein each year of ED experience was associated with increased odds of admission of approximately 1.3.

## 4. Discussion

To varying extents, Australian states are implementing ED-based mental health services to assist emergency clinicians in assessment and management of mental health-related presentations. ED-based mental health services, such as mental health liaison nurses in Victoria, have produced positive outcomes for clinicians, hospitals, and patients, in the form of cost effectiveness [22], reduced self-harm representations [6, 22], reduced waiting times for mental health patients, reduced patient distress, and more efficient coordination of mental health care and therapeutic interventions [23].

Both the Victorian Government and Royal Australian and New Zealand College of Psychiatrists recommend that patients with deliberate self-harm or suicidal ideation not be discharged before a mental health team assessment of the patient has been made [5, 6], ideally in the ED. Despite this, the need for emergency clinicians to possess adequate suicide risk assessment and management skills remains, as these services are not available in some EDs or are available in a more limited capacity [5]. Moreover, the Victorian Government maintains that, regardless of ED-based mental health services, ED clinicians are still expected to take responsibility for and possess skills to appropriately assess and manage mental health-related ED presentations [5].

The growing numbers of patients at risk of suicide attending Australian EDs and the high rates of suicide in Australia highlight this need for front-line clinicians to be able to appropriately assess and manage patients at risk of suicide. Assessment and management of these patients is a complex task for ED clinicians, particularly in the context of the busy ED setting, with significant time constraints limiting in-depth assessments, and a lack of privacy for patients [11].

An initial assessment of the patient's risk to themselves and others, as well as associated protective factors, such as family supports, informs the clinician's disposition decision for the patient, to admit the patient for inpatient services or to refer the patient for review at a later stage. Disposition decisions should consider the patient's preceding risk assessment, legal status under the Mental Health Act, home environment, and potential stressors [4]. Ideally, an emergency clinician would consult an ED-based mental health specialist; however it is recognized that this is frequently not possible due to limited service availability, particularly in smaller hospitals and after hours [5].

Our study found that Australian emergency clinicians were essentially uniform in their disposition decisions following suicide risk assessments across all case scenarios presented. Not surprisingly, the “high risk test scenario” produced the most uniform decision, with the highest percentage of participants choosing to admit the patient. By contrast, the “low risk test scenario” had one of the lowest percentages of participants choosing the modal disposition decision, discharge for community team review within 24 hours; despite a relatively low percentage of participants choosing this modal disposition decision, almost all participants (97.0%) chose to discharge the patient in the dichotomous disposition decision. This was the only scenario in which the modal disposition decision was to discharge the patient. The reluctance of respondents to discharge patients in the case scenarios is consistent with findings from a study of factors affecting ED triage decision making [24] that clinicians have a tendency to make more conservative decisions in difficult or ambiguous presentations.

Scenario 5 produced the least uniform disposition decision. Decisions were divided between choosing to admit the patient and choosing to discharge the patient for community team review within 24 hours. Clinician uncertainty about factors such as the available support to the patient in the Supported Residential Service may have influenced these decisions and may mirror a more general uncertainty about availability and reliability of community support for such people.

Significant associations were found across all five scenarios between the decision to admit the patient described, and higher ratings of the patient's level of risk to self and others and lower ratings of the patient's level of protective factors mitigating risk. Similarly, the decision to admit the patient was significantly associated with higher ratings of participant confidence in all scenarios except the “low risk test scenario,” the only scenario in which the modal disposition was to discharge the patient. This suggests that clinicians perceive admission as a lower risk option and feel more secure with the perceived safety inherent in this higher level of care, consistent with findings in the literature [24]. Although decisions were divided between admitting and discharging the patient for community team review within 24 hours for scenario 5, in which disposition decisions were least uniform, higher levels of confidence were associated with the decision to admit the patient. This perhaps reinforces our perception of lower clinician confidence in the supports available in the community, both in terms of community team review and in the Supported Residential Service where the patient resided in this scenario.

Based on results of this study it is not possible to rule out the influence of clinician factors on disposition decisions, such as years of experience and age since these were significant independent predictors (in two scenarios) after accounting for the influence of risk and protective factors and other demographics. However, the lower frequency and strength of association suggest that these factors often do not play a role in decision making, and when they do their role may be minor relative to risk and protective factors inherent in the presentation, with risk being consistently a significant predictor with odd ratios ranging between 2.6 and 6.1.

Despite results of previous research [15, 16] indicating that Australian emergency clinicians feel that they lack confidence and skills to deal with mental health presentations, clinicians in our study consistently rated their confidence in their disposition decisions as high. This discrepancy may suggest that clinicians lack confidence in other aspects of management besides disposition decision making or may have been the product of the case scenarios presented not being as difficult or complex as real clinical scenarios. Alternatively, the high ratings may be due to the conservative nature of disposition decisions made for the hypothetical scenarios described in the survey, with discharging the patient being the modal disposition decision for only one scenario. This is supported by the significant association between the decision to admit and higher ratings of clinician confidence. The nature of risk assessment in the context of mental health service provision in Australia exposes clinicians and employers to the possibility of litigious proceedings. There is the possibility for conflict between organisational demands and clinical practice where an organisation may wish a clinician to err on the side of caution in the face of good clinical practice [25].

### 4.1. Study Strengths and Limitations

Our study had significant strengths which may be attributed to the research design. Incorporating a pilot study into the research design in which the online tool was validated ensured that the online survey tool used in the main study was coherent and relevant. Modifications to the online survey tool according to results from the validation survey ensured greater participant understanding of online survey items and subsequently ensured that participant responses more appropriately addressed survey items.

The choice of a web-based format for the main survey facilitated inclusion of diverse clinician population groups, as well as maintaining anonymity of respondents. Using the online survey package, “Survey Monkey,” allowed anonymous participation, ensuring greater honesty of responses regarding a subject area in which clinicians have indicated a lack of confidence. The web-based format also facilitated involvement of groups who would otherwise have been difficult to include, improving generalisability of the study findings for a range of ED types. Specifically, the online nature of the survey allowed large numbers of both doctors and nurses to be invited to participate in the research, including those from both metropolitan and regional or rural areas, working in larger and smaller hospitals. Considering the proportionally high levels of suicide in rural areas of Australia, data from clinicians working in EDs in these areas holds particular significance.

This study was the first of its kind to investigate uniformity of ED clinicians' disposition decisions following suicide risk assessments, factors affecting these decisions, and confidence in decisions on a national scale. The inclusion of clinicians from states with varied legislation, ED, mental health and community-based services, patient populations, and clinician training is likely to improve generalisability of the study.

Although a cross-sectional survey was an appropriate choice of study design, this method may have introduced some biases. Selection bias may have been introduced due to the small sample size in the form of volunteer bias [26], wherein characteristics of those volunteering to participate in the research differ from those who do not, leading to sampling error, where the sample is not representative of the population [27]. Emergency clinicians may have been motivated to participate in the research, had they been particularly interested or experienced in the research topic, mental health-related ED presentations, compared to those less interested or experienced. Despite this, there is evidence for the validity of similar research with comparatively low response rates [26]. Although the overall survey response rate was low (7.7%) with 334 eligible participants, the margin of error calculated for the sample was 5.09%, only marginally above the required 5% margin of error.

The length of the survey may have limited sample size. This also resulted in limited collection of demographic information about participants, as demographic questions were at the end of the survey.

The use of case scenario vignettes in the study design allowed a cost-effective uniform delivery of scenarios to a large number of participants, in comparison with studies using a simulated patient in a clinical scenario [28]. However, delivery of scenarios in vignette form did not facilitate a clinician-patient interaction. This limited the ability of the clinician to incorporate nonverbal information gathered about a patient into decision making, one of the three key skills incorporated into King's tool for assessing clinician competency in suicide risk assessment [29]. Our data may have limited generalisability to a real clinical setting; however evidence from literature suggests that vignettes are a valid means for investigating clinical decision making for various patient presentations [18, 19].

Due to the inherently complex nature of mental health assessments and management, this study only investigated uniformity of disposition decisions amongst emergency clinicians, rather than “accuracy” or consistency of decisions. Future studies could consider the possibility of recruiting an expert panel to provide a “best response” for each scenario as a point of comparison with participant modal responses.

## 5. Conclusion

There is considerable uniformity amongst Australian emergency clinicians' disposition decisions following suicide risk assessments. Clinicians tend to choose conservative options and have limited confidence in electing to discharge patients for community management. Clinician ratings of patient risk and protective factors for suicide and confidence in disposition decision are significantly associated with respondent disposition decisions, and perceived risk factors are consistently a strong predictor of disposition decision.

## Figures and Tables

**Table 1:** Summary of disposition decisions, confidence, and risk and protective factor ratings. Data are median (IQR) and modal categorical rating (low, moderate, and high).

Scenario	Disposition decision% (95% CI)	Median rating (IQR) of patient level of risk	Median rating (IQR) of patient level of protective factors	Median rating (IQR) of level of confidence in disposition decision
1	Admit 81.0% (76.0–85.1)	High 8 (7.0–9.0)	Moderate 4 (3.0–6.0)	High 8 (6.0–9.0)
2	Discharge, for community team review within 24 hours, 72.3% (66.7–77.3)	Low 3 (3.0–5.0)	High 7 (6.0–8.0)	High 8 (7.0–8.0)
3	Admit 83.7% (78.7–87.8)	High 8 (7.0–9.0)	Low 3 (2.0–5.0)	High 8 (7.0–9.0)
4	Admit 92.4% (88.3–95.2)	High 9 (8.0–10.0)	Low 3 (2.0–4.0)	High 8 (7.0–9.0)
5	Admit 58.6% (52.2–64.8)	High 7 (6.0–8.0)	Low 3 (2.0–5.0)	High 7 (6.0–8.0)

**Table 2:** Level of self-rated confidence in disposition decision according to decision made.

Scenario		Admit	Discharge	*P* value*
1	Median rating confidence in disposition decision	8 (IQR 6.0–9.0)	6 (IQR 4.8–8.0)	<0.001
2	Median rating confidence in disposition decision	7.5 (IQR 7.0–8.0)	8 (IQR 7.0–8.0)	0.871
3	Median rating confidence in disposition decision	8 (IQR 7.0–9.0)	7 (IQR 5.0–8.0)	<0.001
4	Median rating confidence in disposition decision	8 (IQR 7.0–9.0)	6 (IQR 4.8–7.0)	<0.001
5	Median rating confidence in disposition decision	8 (IQR 6.0–9.0)	6 (IQR 5.0–7.0)	<0.001

*Mann-Whitney *U* test.

**Table 3:** Factors predicting disposition decisions.

		Sig.	OR	95% C.I. for OR	
				Lower	Upper
Scenario 1	Risk rating*	**0.002**	**2.754**	**1.468**	**5.167**
	Protective factor rating*	0.062	0.557	0.301	1.031
	Staff age*	0.980	1.001	0.931	1.076
	Years in ED*	0.354	1.041	0.956	1.133
	Staff gender	0.798	1.127	0.450	2.827
	ED type	0.575	0.762	0.295	1.968
	Staff role	0.494	0.710	0.266	1.895
	Constant	0.019	0.014		

Scenario 2	Risk rating*	**0.002**	**2.754**	**1.468**	**5.167**
	Protective factor rating*	0.062	0.557	0.301	1.031
	Staff age*	0.277	1.112	0.918	1.348
	Years in ED*	0.476	0.926	0.749	1.144
	Staff gender	0.798	1.355	0.133	13.782
	ED type	0.232	0.233	0.021	2.547
	Staff role	0.109	17.066	0.529	550.462
	Constant	0.025	0.000		

Scenario 3	Risk rating*	**0.000**	**2.645**	**1.796**	**3.895**
	Protective factor rating*	**0.003**	**0.677**	**0.522**	**0.880**
	Staff age*	**0.043**	**0.919**	**0.848**	**0.997**
	Years in ED*	0.596	0.975	0.888	1.071
	Staff gender	0.271	1.871	0.613	5.711
	ED type	0.849	1.110	0.382	3.223
	Staff role	0.192	0.468	0.150	1.464
	Constant	0.903	1.261		

Scenario 4	Risk rating*	**0.000**	**6.063**	**2.839**	**12.950**
	Protective factor rating*	0.940	0.983	0.627	1.542
	Staff age*	0.075	0.882	0.769	1.013
	Years in ED*	**0.040**	**1.266**	**1.011**	**1.585**
	Staff gender	0.398	2.251	0.342	14.791
	ED type	0.933	0.924	0.147	5.798
	Staff role	0.245	0.249	0.024	2.591
	Constant	0.043	0.001		

Scenario 5	Risk rating*	**0.000**	**4.040**	**2.656**	**6.145**
	Protective factor rating*	**0.001**	**0.688**	**0.550**	**0.860**
	Staff age*	0.757	0.989	0.920	1.063
	Years in ED*	0.519	1.029	0.943	1.124
	Staff gender	0.079	2.203	0.913	5.313
	ED type	0.942	1.033	0.425	2.515
	Staff role	0.842	0.914	0.377	2.213
	Constant	0.000	0.000		

*Continuous variable.

Outcome measure coded as 0: discharge, 1: admit. Statistical significance printed in boldface.

**Table 4:** Scenarios presented to survey respondents.

Scenario one described Trevor, a recently retired 68-year-old male, involved in a car accident under the influence of alcohol. His history included recent depression with GP management, increased alcohol intake, and discussions about suicide with his wife.	

Scenario two was the low risk test scenario and described Mary, a 22-year-old female who lives with her mother, presenting to ED after cutting herself following an incident with a coworker. Since seeing a counsellor, her self-harming had been happening less often.	

Scenario three described Aaron, a 35-year-old male with Down's syndrome, brought into ED by his mother, who was reluctant to have him admitted. Aaron had been hitting his head against a brick wall, saying that his recently deceased father was telling him to kill himself. He had been diagnosed with depression but refused medication.	

Scenario four was the high risk test scenario and described Gayle, a 39-year-old mother of four young children, with a history of childhood sexual abuse, substance abuse, and psychiatric issues. She was brought into ED after her eldest child found her under the influence of alcohol with a noose.	

Scenario five described Alan, a 36-year-old man, with a history of schizophrenia, persistent delusions, and recent medication noncompliance and negative thoughts. He lived in a supported residential service (SRS), where he had been stockpiling his medication with plans to overdose, and presented to ED with his new case manager.	
